# A European approach to clinical investigator training

**DOI:** 10.3389/fphar.2013.00112

**Published:** 2013-09-09

**Authors:** Jean-Marie Boeynaems, Cindy Canivet, Anthony Chan, Mary J. Clarke, Catherine Cornu, Esther Daemen, Jacques Demotes, Katelijne De Nys, Barry Hirst, Ferdinand Hundt, Behrouz Kassai, Sandor Kerpel-Fronius, Lucy Kiessig, Heinrich Klech, Jean-Pierre Kraehenbuhl, Pierre Lafolie, Martin Lucht, Detlef Niese, Christiane Pauli-Magnus, Barbara Peters, Ralf Schaltenbrand, Armel Stockis, Martina Stykova, Nicolette Verheus, Ingrid Klingmann

**Affiliations:** ^1^Erasme Hospital, PHARMED, Université Libre de BruxellesBrussels, Belgium; ^2^F-CRINToulouse, France; ^3^Pfizer Healthcare IrelandDublin, Ireland; ^4^Pfizer USADetroit, MI, USA; ^5^Centre d'Investigation Clinique, Hospices Civils de LyonLyon, France; ^6^Association of Clinical Research ProfessionalsAlexandria, VA, USA; ^7^European Clinical Research Infrastructure Network & Institut National de la Santé et de la Recherche MédicaleParis, France; ^8^Katholieke Universiteit LeuvenLeuven, Belgium; ^9^University of NewcastleNewcastle, UK; ^10^University of Duisburg-EssenEssen, Germany; ^11^Université Claude Bernard Lyon 1Lyon, France; ^12^Semmelweis UniversityBudapest, Hungary; ^13^Johnson and JohnsonSlough, UK; ^14^Medizinische Universität WienVienna, Austria; ^15^Health Sciences eTrainingEpalinges, Lausanne, Switzerland; ^16^ECRIN, Karolinska University HospitalStockholm, Sweden; ^17^University of FreiburgFreiburg, Germany; ^18^Novartis Foundation for Medical-Biological ResearchFreiburg, Germany; ^19^University of BaselBasel, Switzerland; ^20^Clinical Trial Unit, University of BaselBasel, Switzerland; ^21^UCB PharmaBraine-l'Alleud, Belgium; ^22^DIABasel, Switzerland; ^23^Johnson and JohnsonAmsterdam, Netherlands; ^24^European Forum for Good Clinical PracticeBrussels, BelgiumAll the authors have been members of PharmaTrain & ECRIN Advisory Group on clinical investigator training

**Keywords:** clinical trials as topic, clinical investigator, training, GCP, certification

## Abstract

A better education and training of clinical investigators and their teams is one of the factors that could foster the development of clinical research in Europe, a key objective of the Innovative Medicines Initiative (IMI). PharmaTrain (an IMI programme on training in medicines development), and European Clinical Research Infrastructures Network (ECRIN) have joined forces to address this issue. An advisory group composed of representatives of universities, pharmaceutical companies and other organisations met four times between June 2011 and July 2012. This resulted in a position paper proposing a strategy to improve and harmonize clinical investigator training in Europe, and including a detailed syllabus and list of learning outcomes. Major recommendations are the establishment of minimal and mutually recognized certification requirement for investigators throughout the EU and the creation of a European platform to provide a suitable course and examination infrastructure.

## The need for improved and harmonised investigator training in europe

Planning, preparing and organising clinical trials at the investigator site has become a highly complex task taking into consideration the need to protect the patients, to generate reliable data, to perform the trials efficiently, with increasingly short timelines, and to fulfill all quality requirements according to the current legislation and inspection requirements. Obviously, it is important that European sites are able to perform clinical trials according to the required standards. While the number of performed clinical trials has only slightly decreased in the last years, other regions like Eastern Europe, Asia and South America have become more attractive to biopharmaceutical industry sponsors, leading to a decreased number of studies they initiate in Western- and Central Europe. Overall, the number of clinical trials in Europe has decreased by 20% in the last 3 years^1^ ^2^ ^3^ ^4^ ^5^. The complexity of clinical trials and the regulatory requirements have increased significantly in the last few years, requiring an increasing level of scientific, methodological, regulatory and organisational know-how to be able to perform clinical trials efficiently. Despite pharmaceutical industry's increase of the percentage of multi-national trials and the number of sites involved, the number of enrolled patients per year has not increased (EUdraCT database), making the planning and execution of clinical trials difficult and costly. In Europe, clinical research (clinical trials science and methodology) is often not highly regarded academically and collaboration with pharmaceutical industry in performing the trials is still subject to suspicion of commercial bias and perceived lack of academic credibility, as presented in a European Science Foundation (ESF) report on investigator-driven clinical trials^6^. The most important recommendation of this report to reverse this trend was: To improve the education, training and career structure and opportunities for scientists involved in patient-oriented clinical research.

The principal clinical investigator has a crucial role in the clinical trial performance at the site. Clinical investigators are physicians who, in most cases, primarily perform diagnosis and treatment of diseases of patients under their care. Medicines under clinical investigation and the organisation of clinical trials are only a small part of their daily hectic and usually overloaded clinical activities. However, having the skills and infrastructure to enrol more patients makes the performance of a trial more worthwhile in every aspect. Yet, there is still broad lack of understanding amongst investigators about the benefits of training in efficient and reliable clinical trial performance.

All parties involved in organising and supervising clinical research agree that investigators need adequate training to carry out their duties. In particular this is stated in the International Conference on Harmonisation (ICH) Guideline for Good Clinical Practice (GCP) and in the European Clinical Trials Directive 2001/20/EC. Large biopharmaceutical companies have developed their own GCP courses as a minimal requirement and demand that every investigator attends these courses as a prerequisite to become an investigator in their clinical trials. The current qualification standards for investigators are generally vague and vary widely between European Union (EU) countries. Only in Sweden, UK, Switzerland, Hungary, and Lithuania a GCP certificate is a minimum regulatory requirement to participate in clinical trials. In some EU countries, like Germany or Italy, ethics committees expect to see a GCP certificate as a demonstration of investigator suitability. However, training over one or two days in GCP does not enable physicians to thoroughly comprehend their role in protecting trial participants and to generate quality data in an efficient way in all types of studies. Moreover most ethics committees in Europe are satisfied with a curriculum vitae documenting clinical credentials in the respective therapeutic area.

Clinical research is only very marginally subject to undergraduate medical training in Europe, and there is no post-graduate education obligation for physicians performing and taking responsibility for clinical trials in most EU countries. Clinical trial units in hospitals, private training providers and some universities (e.g., Basel, Copenhagen, Leuven…) across Europe have started to offer comprehensive investigator training to improve the competence of their investigators and the number of clinical trials successfully performed in their hospitals. However, these are local, spontaneous and often reactive activities. There is a need to develop a strategy for investigator training in Europe that

- is demonstrably able to enhance efficiency and reliability of investigator activities
- can be applied in all EU countries, fulfilling regulatory and ethics committee requirements
- fulfils pharmaceutical industry's quality expectations
- follows a syllabus that covers the full spectrum of investigator activities, not just the GCP basics
- is adapted to investigators' respective roles and responsibilities in a clinical trial
- can be integrated into the investigators' work schedule
- is provided by demonstrably qualified training organisations
- ensures demonstration of achieved learning outcomes
- is financially affordable
- can be performed without undue investment of investigators' time.

The here proposed strategy for investigator training will further improve the credibility of clinical research and increase patient throughput in clinical studies, a key objective of the Innovative Medicines Initiative (IMI).

## Pharmatrain and ecrin

PharmaTrain is one of the IMI Education and Training projects and focusses on the development of a comprehensive training infrastructure in the area of pharmaceutical development which includes the areas of pharmaceutical medicine, regulatory affairs and clinical trial performance. The project is based on the creation of a network of Diploma and Master Courses in pharmaceutical development and in regulatory affairs, located in many different countries. The PharmaTrain consortium partners, consisting of universities, not-for-profit organisations and pharmaceutical companies, have developed and implemented an advanced standard for content and teaching methodology based on an agreed syllabus and curriculum and including e-learning opportunities. PharmaTrain has established a European quality management infrastructure to assess quality, content and performance of the associated courses, an infrastructure to manage the examination process in all associated courses and an IT platform within EMTRAIN's “on-course” database to enable easy identification of the most suitable training opportunities.

ECRIN, the FP7-funded European Clinical Research Infrastructures Network, is a sustainable, not-for-profit infrastructure supporting multinational clinical research projects in Europe. ECRIN provides information, consulting and services to academic investigators and sponsors in the preparation and in the conduct of multi-national clinical studies, for any category of clinical research and in any disease area. This is particularly relevant for investigator-initiated (academic, non-industry sponsored) or small and medium enterprise-sponsored clinical trials and for clinical research on rare diseases where international cooperation is a key success factor. ECRIN is based on the connection of coordinating centers for national networks of clinical research centers and clinical trials units, able to provide support and services to multinational clinical research. Relevant tools for clinical researchers involved in multinational clinical trials are available on the ECRIN website.

Based on global and other regions' activities to increase investigator competence like e.g., the Organisation for Economic Co-operation and Development (OECD), Academy of Pharmaceutical Physicians and Investigators (APPI), or Alliance for Clinical Research Excellence and Safety (ACRES) initiatives, there is an increasing wish to harmonize investigator training requirements in the EU. However, at the same time it will be vital to build an infrastructure that gives investigators easy and affordable access to training and examination.

PharmaTrain and ECRIN have joined forces to utilize their European reach and impact on clinical research to promote and establish such a European investigator training infrastructure leading to a clinical investigator certificate (CLIC). They invited other organizations involved in investigator training to join an Advisory Group in order to work out a suitable strategy.

## Different levels of competence

Not all professionals involved in clinical research need to acquire the same level of competence in clinical trial performance. In fact, some are only involved in the study site team. Others assume responsibility as principal investigators. Still others may consider clinical research as a major component of their professional life and even initiate new studies on sponsor level. A somewhat different combination of knowledge and skills is required for investigators performing Phase I trials in a dedicated unit. A training approach adapted to the training needs in human pharmacology studies might be initiated in near future by experts in that field.

To date a concept of 3 levels of competence has emerged. It is found in the Swissmedic (Swiss Agency for Therapeutic Products) guidelines^7^, which distinguish sub-investigator, investigator and sponsor-investigator, as well as in the USA-based APPI Statement of Clinical Investigator Competence^8^, which relates them to the depth of commitment to clinical research.

It is therefore recommended that courses offer the option of different levels of training related to distinct responsibilities in the performance of clinical trials:

- Level 1: site staff.
- Level 2: (principal) investigator (responsibility for a clinical trial at a site).
- Level 3: sponsor-investigator (overall responsibility for a clinical trial).

These 3 levels represent increasing competence and individuals may move from one level to an upper level over time.

Level 1 encompasses a basic core of knowledge that is common to the various professionals in a study team involved in the preparation and conduct of studies at investigational sites. The target audience is therefore constituted by medical (sub-investigator) and non-medical (study nurse, study coordinator, study manager…) staff.

Level 2 represents the knowledge in regulatory and managerial aspects of a clinical trial requested from a principal investigator according to ICH-GCP definition, EU and national legislation.

Level 3 corresponds to investigators for whom clinical research is a major element of their professional life and who assume responsibility for investigator-initiated trials. The term “sponsor-investigator” is not optimal since in general the sponsor is an institution (e.g., an hospital or university) and not an individual, but in contrast to “trial-initiating investigator,” “sponsor-investigator” expresses the type of knowledge such type of investigator needs to have.

These 3 levels also differ by their national vs. international dimension. The content of the courses will include national regulations at level 1, an overview of the international regulatory environment at level 2 and a detailed knowledge of EU and international regulations at level 3.

To demonstrate the acquired level of knowledge required to receive the respective Investigator Training Certificate, it is recommended to require a mandatory examination.

## Content and learning outcomes of clinical investigator courses

This question has been addressed in several documents:

- A European syllabus for Training Clinical Investigators^9^.
- Swissmedic guidelines defining the training content requested for a sub-investigator, investigator and sponsor-investigator respectively^7^.
- The APPI Consensus Statement^8^.
- The IMI PharmaTrain Manual^10^ defining the contents and learning outcomes of courses in pharmaceutical medicine/medicines development sciences, of which clinical research is an essential component.

Based on these previous documents, a syllabus and learning outcomes have been defined for each of the 3 levels defined above (Tables 1–3). The course content is presented incrementally, i.e., the level 3 syllabus and learning outcomes only contain the elements to be taught in addition to those of levels 1 and 2. This syllabus is currently focused on medicinal products and should later be expanded to other areas of research such as medical devices.

## Format and duration of the courses for clinical investigators

It will be left to the student to decide on the number of hours he/she will invest into learning and the modus of learning. Courses for clinical investigators can be entirely face-to-face or involve mostly e-learning complemented by a short face-to-face session. The PharmaTrain e-library includes a course for clinical investigators in which the content has been stratified according to the 3 levels of competence, mentioned earlier^11^. There is a broad offering of investigator training options by many different organisations in different countries. These existing resources should be used, provided they are in line with the above mentioned syllabus and learning outcomes.

At level 1 and 2, the courses could be given in the local language or in English, whereas English is mandatory for level 3.

The optimal duration of a clinical investigator course will depend on the targeted level of competence. The ESF syllabus suggested a 3–5 days training duration^9^. Although these figures are approximate (especially for e-learning) and not mandatory, the following durations are recommended:

- 2 days (16 h) for level 1.
- 5 days (40 h) for level 2, that is a 3 days increment as compared to level 1.
- 8 days (64 h) for level 3, that is a 3 days increment as compared to level 2.

Beside these basic courses, there is also room for more extensive courses such as full master programmes in clinical research, or diploma courses in clinical trial practices and management that are organised in some European countries.

A grandfathering clause is proposed for a duration of 3 years after implementation, allowing that the established investigators will be eligible to receive the certificate without passing the examination, provided that they sit the examination in form of self-control and submit a detailed CV, including the list of clinical trials in which they have been involved.

A Continuous Professional Development (CPD) programme requiring the attendance to short refresher/update courses, at least every 3 years, should also be established.

## Course accreditation

As part of PharmaTrain's network of course providers in pharmaceutical medicine/medicines development sciences, a European network of CLIC course providers will be established. The course providers will be accredited by the PharmaTrain Central Office according to the agreed and implemented PharmaTrain quality standards.

PharmaTrain will establish a “CLIC Course Recognition” award for those courses fulfilling the PharmaTrain quality criteria and syllabus/learning outcome requirements.

It is proposed to develop a database in the PharmaTrain Central Office containing the accredited courses as well as the contact details of those certified course participants who gave permission to keep their personal data in order to remind them of their CPD needs.

## Examination and certification

It is proposed that one obligatory face-to-face day encompassing the examination will be required as pre-requisite for the certificate. Examinations will be organized at a local level by course providers. Candidates who have not attended courses will be allowed to sit these examinations. These will consist of a multiple choice questionnaire (MCQ) involving the physical presence of candidates in order to be sure of their identity; this could change with time in relation with technological evolution. Distinct questions will be proposed for the different levels of competence required. The MCQs should include at least 50 questions (level 1), 60 questions (level 2), or 80 questions (level 3).

It is proposed to establish a pool of ca. 500 questions for the 3 levels, subject to a continuous improvement process and sensitivity analyses. The questions will be made publicly available (“drivers-licence approach”).

The course/examination providers will request 80% of the questions from the PharmaTrain Central Office, whereas 20% of the questions can be related to national legislation/regulations and be prepared by the local examination/course providers.

At levels 1 and 2, the examination might be in national language rather than English; national course providers will be encouraged to provide the translation of the questions to the central question pool.

Management and scoring of the examinations will be performed by a central unit, according to the Good Examination Guidelines established by PharmaTrain. Answers will be entered on a computerised sheet and automatically evaluated by the central unit selected by the PharmaTrain Central Office, to ensure objectivity and to maintain standards and comparability of scoring.

Certificates will be issued preferably by national universities. If this is not achievable in a country then issuing by national physician associations or nation-wide academic institutions should be sought.

The certificates will be delivered by the course provider containing the university stamp, the PharmaTrain and ECRIN labels and the course provider's label. The certificates will mention the level of competence and all nationally required information.

## Conditions of success and impact

The development of a European programme of clinical investigator training and certification aims at further increasing the quality of clinical trials in Europe, in order to support the faster discovery and development of better medicines for patients and to enhance Europe's competitiveness, which are the goals of the IMI. At the end of the day the impact of investigator training on the quality of clinical research will need to be evaluated using qualitative and quantitative indicators, such as a decrease in the number of observations during audits and inspections.

Success depends on the full cooperation and involvement of investigators, sponsors, ethics committees, medical associations and regulatory authorities. In particular the pharmaceutical sponsors should view clinical investigator training as a new opportunity to enhance quality and efficiency, with the ultimate aim of accruing more patients data in European countries and facilitating EU regulatory filings. These training courses should reduce/obviate the need for sponsor-organised GCP training sessions that cover only part of the training needs and lead to a needless redundancy. Sponsors should recommend and motivate their investigators to attend formal training courses to obtain formal certification. Ethics committees and regulatory authorities should aim at harmonizing regulations and creating a minimal and mutually recognised certification requirement for investigators throughout Europe.

## Recommendations

The following steps are recommended to improve and harmonize clinical investigator training and certification in Europe.

- EU and national regulatory authorities should harmonize regulations and create a minimal and mutually recognized certification requirement for investigators throughout the EU.
- Pharmaceutical sponsors should reduce the need for redundant GCP training sessions that cover only part of the needs and motivate instead investigators to attend training courses and obtain formal certification.
- A European platform should be created to provide a suitable course and examination infrastructure, assess courses and harmonise examinations throughout the EU in order to improve quality and comparability. That platform should be based on current and past European projects in the field such as IMI PharmaTrain and ECRIN.

## Conflict of interest statement

The authors declare that the research was conducted in the absence of any commercial or financial relationships that could be construed as a potential conflict of interest.

## Footnotes

^1^European clinical trial rules under fire, Nature, March 2009, doi:10.1038/news.2009.163

^2^Managing clinical research in the UK, Warwick Business School, December 2009, http://www.qmul.ac.uk/docs/research/65670.pdf

^3^UK doctors demand research reform, Nature, May 2010, doi:10.1038/news.2010.243

^4^Clinical research footprint and strategic plan to promote clinical research in Belgium, pwc, June 2012, http://pharma.be/assets/files/3229/3229129860438902421759.pdf

^5^Europe faces decline in clinical trials activity, Public Service Europe, March 2013, http://www.publicserviceeurope.com/article/3240/europe-faces-decline-in-clinical-trials-activity

^6^Forward Look-Investigator-driven clinical trials, European Science Foundation, March 2009, http://www.esf.org/fileadmin/Publicdocuments/Publications/IDCT.pdf

^7^Requirements for the training of co-investigators, principal investigators and sponsor-investigators involved in clinical trials on therapeutic products, http://www.swissmedic.ch/bewilligungen/00089/01100/index.html?lang=en

^8^Statement of clinical investigator competence, APPI Consensus Statement, Monitor, August 2011: 79–82

^9^A European Syllabus for Training Clinical Investigators, European Science Foundation, July 2003, http://www.esf.org/publications/medical-sciences.html?txccdamdlpi1%5Bpointer%5D=1&cHash=5facba551e13516e3a4b9154f8680f35

^10^PharmaTrain Manual Curriculum Standards and Best Practices, June 2013, http://www.pharmatrain.eu

^11^eCLIC, http://www.pharmatrain.eu or http://clic.bio-med.ch/cms/Default.aspx

