# Supplementary material for: A European approach to clinical investigator training
Source: Front Pharmacol. 2013 Sep 9;4:112. doi: 10.3389/fphar.2013.00112 (PMC3766792; doi:10.3389/fphar.2013.00112)
Supplement: Supplementary file 1 [file DataSheet1.DOC]

**Table 1 Clinical Investigator Training – Level 1**

| **Topics** | **Content** | **Learning Outcomes** | **Duration (h)** |
| --- | --- | --- | --- |
| Introduction to the ethics of clinical research and Good Clinical Practice | - History and justification of the regulations for subject protection - Origin and principles of ICH-GCP - Responsibilities of the various players | - Recognise the impact of historical events that have contributed to the current regulations and guidelines - State the documents that have contributed to current regulations and guidelines - Describe the objectives and role of the International Conference on Harmonisation - Explain the impact of the principles of ICH-GCP - Describe the roles of Sponsor, Monitor, Investigator, Ethics Committee, Regulatory authorities - Summarise the rights of subjects in clinical trials and the role of the investigator in protecting them - Define fraud and misconduct in clinical research | 1 |
| Overview of the medicine development process | - The various steps of the medicines development process: sequence and duration | - Understand the various phases of medicines development, their timelines and attrition | 1 |
| Introduction to clinical research methodology | - Definition of the phases of clinical development (I-IV) and related research objectives - Structure of a clinical trial - Key elements of trial design - Definitions of parallel groups versus crossover, control, placebo, randomisation, blinding, bias, intention-to-treat | - Define the research questions to answer and types of endpoints in each clinical trial phase - Define the subject population, size and duration of trials in each phase - Explain the design aspects of a Randomised Controlled Trial - Describe the different periods a clinical trial may contain from identification of the subject until his/her last visit - Describe the different types of study populations in the statistical evaluation | 1 |
| Legislative framework and guidance for clinical research | - International regulatory environment - Applicable national regulations | - Explain the link between ICH-GCP, EU Directives/Regulations and national regulations - Describe how EU Directives impact on national regulations - Explain the key national legal requirements for clinical trials in your country | 1 |
| Planning and preparation of a trial | - Review of protocol and related material - Interactions between investigator and sponsor (pre-study visit, investigator selection, budget and contract, initiation visit) - Submission to the ethics committee - Submission to national regulatory authorities - Preparation of study-related processes and documentation | - Explain how to explore the suitability of the indication and protocol implications for your site - Understand the need of an effective communication between the various stakeholders (sponsor, investigative team members, ethics committee, regulatory authorities, hospital administration, patients, treating physicians) - Order key events in the conduct of a clinical trial - Define the key dossier elements and timelines for national ethical review - Define the key dossier elements and timelines required for study approval by the national competent authorities - Understand the protocol-required site processes - Explain how to prepare the required study documentation together with the sponsor | 2 |
| Site organisation and management | - Evaluation of resources needed for the clinical trial - Organisation of the investigative site team - Organisation of a patient visit | - Describe the process of defining the staff level, staff time and facilities required for a clinical trial - Explain the activities required to prepare the investigative site team for the study initiation visit - Explain the activities required to organise a patient visit | 2 |
| Subject recruitment, enrolment and retention | - Challenge and strategy of recruitment - Different phases of recruitment and enrolment - Patient information and informed consent process in adults and children - Randomisation in practice - Compliance check - Subject retention - Personal data confidentiality, patient privacy | - Understand the challenge of enrolling the required number of subjects in the given timelines - Describe options for finding study subjects including advertisement requirements - Define screening, recruitment and enrolment - Define the informed consent process for adults and for children/parents and how it is documented - Recognize the importance of understandable, complete and honest information for the patient’s autonomous decision on participation in the study before any trial-related procedure - Identify the people that may be involved in the informed consent process and recognize what can be delegated by the principal investigator - Explain the differences between consent withdrawal, patient withdrawal and early study termination - Describe the randomisation process in open and blinded studies - Describe the options to identify and supervise patients’ compliance - Describe ways to improve subject retention - Recognize the patients’ rights on privacy and data protection and describe best practices to achieve this | 2 |
| Overview of in-trial procedures | - Source documents and essential documents - Subject visits, measurements and assessments - Completion, correction and control of the Case Report Form - Management of the investigational product - Monitoring visits - Trial close-out | - Understand the difference between essential and source documents - List the main documents that must be in place prior to randomization of the first subject - Recognize the responsibility of the investigative team in collecting complete, accurate and traceable data - Describe the aspects required to ensure quality measurements and assessments - Define the different types of data and data sources in a clinical trial - Describe the technical options for case report forms, the process and staff involved in case report form completion and correction - Understand the consequences of missing data - Understand the role of the investigative team in the management of the investigational medicinal products and the differences with routine medicines prescription - Describe the activities required for correct study medication handling - Describe the process of drug accountability - Understand the basic aspects of biological samples management - Define the types of monitoring visits - Describe the site’s activities required for preparation, execution and follow-up or a monitor’s visit - Understand the requirements for storage and archival of essential documents at the investigational site - Describe the site’s activities required to study close-out | 4 |
| Introduction to safety | - Basic definitions and classification of adverse events (AE, SAE, ADR, SUSAR…) - Reporting and management of adverse events, including un-blinding - Emergency situation handling | - Recognize that the on-going collection of safety data is a regulatory requirement and allows adequate evaluation of the risk/benefit ratio - Match key safety terms and abbreviations to the appropriate definition - Understand the difference between adverse event and adverse reaction - List the criteria defining a serious adverse event - Understand the difference between serious and severe events - Describe the process of identification, adequate reporting and management of serious adverse events - Understand the implications of breaking the blind and list circumstances where un-blinding is justified - Understand the need for continuous emergency situation training of the site staff and the control of the emergency equipment | 1 |
| Quality assurance, monitoring, audits and inspections | - Basic concepts in quality management (Quality assurance incl. SOPs, quality control… - Monitoring versus audits versus inspections - Audit and inspection findings | - Recognize the importance of quality management in clinical trials - Describe the elements of a quality management system like quality assurance, quality control, training , CV and job description(s), and define the difference between quality control and quality assurance - Define the difference between monitoring, audits and inspections - Describe potential outcomes/consequences of audits and inspections | 1 |
