# Supplementary material for: A European approach to clinical investigator training
Source: Front Pharmacol. 2013 Sep 9;4:112. doi: 10.3389/fphar.2013.00112 (PMC3766792; doi:10.3389/fphar.2013.00112)
Supplement: Supplementary file 2 [file DataSheet2.DOC]

**Table 2 Clinical Investigator Training – Level 2**

Contents and learning outcomes of Level 1 (Table 1) +

| **Topic** | **Contents** | **Learning outcomes** | **Duration (hrs)** |
| --- | --- | --- | --- |
| Basic concepts for designing and evaluating clinical trials | - Basic statistical concepts and definitions (confidence interval, statistical significance, odds ratio…) - Types of study (observational versus experimental) and level of proof - Types of design (inter-patients, intra-patients, sequential) - Types of comparison (superiority, non-inferiority…) - Various types of bias and measures to avoid them - Sample size calculation - Types of analysis (intention-to-treat versus per protocol) - Meta-analysis and evidence-based medicine - Subgroups and post-hoc analyses - Statistical significance and clinical interpretation | - Match basic statistical terms with their definition - Describe basic statistical principles and methods for clinical data analysis and reporting - List the major types of bias in clinical trials - Identify the benefits of randomization as a means to reduce bias and confounding - Justify the use of blinding to minimise bias - Describe the information required to calculate the sample size - Understand the difference between intention-to treat and per protocol analysis - Explain the principles and limitations of meta-analyses - Evaluate the strength of evidence of clinical trials - Describe the limitations of clinical trials in predicting effectiveness - Define the rules for subgroups and post-hoc analyses - Explain the meaning of “statistical significance” and its relevance for clinical interpretation | 3 |
| Study protocol | - Structure and contents - Objectives and endpoints - Inclusion/exclusion criteria - Study diagram and flowchart - Measurements and assessments - Protocol amendments | - List the major sections of a protocol - Understand the relevance of primary and secondary objectives and endpoints for the performance of the study at site - Understand the practical implications of adherence to in- and exclusion criteria for subject recruitment - Understand the usefulness of study flowcharts for the management of the study - Describe how to ensure optimal execution of the study activities described in the flowchart during a subject’s study visits at the site - Describe how measurements and assessments for clinical trial subjects according to a protocol differ from routine measurements and assessments - Describe the practical implications of a protocol amendment | 2 |
| Ethics of clinical research | - Investigator responsibilities - Criteria for the ethical evaluation of studies (scientific validity, equipoise…) - Risk-benefit assessment - Ethical review procedures - Use of placebo - Follow-on treatment - Conflicts of interest - Misconduct and fraud - Publication bias and clinical trial registries | - Describe the investigator’s responsibility for the study subjects’ safety, integrity and well-being - Acknowledge the investigator’s responsibility for fully knowing the scientific background of the study and clinically relevant aspects of the study medication - Understand the investigator’s responsibility for the selection of studies with acceptable benefit/risk ratio and suitability for his/her site - Explain the ethical problems in placebo-controlled studies - Understand the need for transparency in study organisation and fair access to study medication - Describe the investigator’s role in ensuring adequate follow-on treatment for study participants - Identify the areas of investigators’ potential conflicts of interest - Summarize the role an ethics committee assumes to protect the rights and safety of subjects - Define the criteria used by an ethics committee for approving a study - Describe the documentation that must be received from the ethics committee before starting a study - List the minimum on-going communication required by ethics committees - List indicators that could lead to suspect fraud or misconduct - Recognize the implications of confirmed fraud or misconduct in a clinical trial - Understand the need to register a study in a publicly accessible registry - Recognize the need and difficulties in publishing every study, independent of its outcome | 2 |
| Informed consent process | - Right of subjects - Information transmission and understanding of the subject - Re-consent | - Acknowledge the investigator’s responsibility for ensuring an adequate informed consent process and documentation - Explain the requirements for appropriate language when writing a patient information sheet and informed consent form - List instances in which a subject cannot provide informed consent - List the characteristics required from a witness - Explain the difference between and conditions for “consent” and “assent” - Define the need and process for including a legal representative in the informed consent process - Explain your national requirements for informed consent in emergency situations - Appreciate that informed consent is an on-going process | 1 |
| Introduction to clinical studies in special and vulnerable populations | - Children - Elderly subjects - Incapacitated adults - Pregnancy and breast-feeding - Orphan diseases | - Identify vulnerable patient groups and the methods used to protect them - Understand why these populations are considered vulnerable - Understand the purpose and content of the Paediatric Investigation Plan - Acknowledge the additional complexities in managing studies with children, elderly, mentally handicapped or unconscious patients - Recognize the special protection needs for pregnant or breast-feeding women in clinical trials | 1 |
| Document management | - List of the essential documents generated before, during and after the trial - Investigator site file - Rules for archival (investigator versus sponsor file, duration) | - List all essential documents that must be in place prior to randomization of the first subject - Identify all documents required to be in the Investigator Site File at study end - Define the site documents that are not supposed to go into the Trial Master File - Understand appropriate document management, including corrections and version control - Recognize that investigators are responsible for the archival of essential documents for the time period defined by local regulations or longer if required by the sponsor | 2 |
| Safety data | - AE collection and assessment - SAE assessment and reporting | - Describe the process for reliable adverse event collection, qualified assessment, complete documentation and adequate reporting - Describe the elements of a CIOMS form - Explain the rules for causality and expectedness assessment | 2 |
| Insurance issues | - Various types of insurance contracts and coverage - Variability of insurance regulations between countries | - Understand the concept of no-fault liability - Acknowledge the need for the investigator’s medical mal practice insurance and a study-related subject liability insurance - Describe the national subject insurance requirements | 1 |
| Management of the investiga-tional medicinal product | - Types of medication in a study - Packaging and labelling - Storage and handling - Return and accountability - Compliance monitoring | - Define the different types of medication administered to study subjects and the respective financial coverage conditions - Understand the process and timelines for study medication preparation including stability timelines, blinding, labelling and packaging - Identify the investigator’s responsibilities for appropriate study medication handling and accountability - Acknowledge the need for respecting and controlling IMP storage conditions - Appreciate the importance of seamless documentation of accountability for IMP from receipt to return or destruction - Explain the investigator’s potential interaction with the pharmacy - Recognize the investigator’s responsibility for subjects’ reliable medication compliance | 1 |
| Biological samples management | - Use of biological markers for patient selection and evaluation of efficacy and safety - Shipment requirements - Archival in biobanks | - Identify the specific role of biological outcomes - Recognize the potential need for additional informed consent when samples are taken for genetic analysis - Respect that stored biological samples can only be used for the purposes described in the protocol and the informed consent - List the reference documents for the management of biological samples - Acknowledge the investigator’s responsibility for sampling, work-up, storage and shipment of biological samples - Understand the principles of biobanking including the rules for anonymisation of samples | 1 |
| Data collection and management, final reporting | - Structure of the CRF - Data collection and documentation process - Central monitoring and quality control, data queries - Advantages and disadvantages of electronic data capture - Confidentiality and data protection - Final reporting | - List the key elements of a CRF - Explain the standard data collection, documentation, control and investigator review process - Understand that the reliability of study results is based on the completeness, consistency and correctness of the data provided by the investigator in the CRF - Explain the importance of maintaining an audit trail - List the advantages and disadvantages of electronic data capture over paper CRF - Understand the difference between the source documents and the CRF - Explain the key confidentiality rules between investigator and sponsor as well as investigator and subject - Describe the process of subject data pseudonomisation and anonymisation - Explain the investigator’s role and support to final reporting of the study | 2 |
| Clinical project management | - Adequate resources and facilities - Project planning - Screening, recruitment and retention - Management of deviations and mistakes - Interaction with monitors, auditors and inspectors - Communication - Quality management - Training - Supervisory committees | - Explain the process to ensure suitable staffing, medical competence and facilities in all departments involved in the study at this site - Describe the study planning process for this site - Recognize the options for delegation of responsibilities for screening, enrolment and medical care of subjects - Explain how to ensure adequate emergency coverage during a study - Describe how to identify and handle mistakes, deviations and omissions in a study - Describe investigator tasks, responsibilities and delegation options in interaction with monitors, auditors and inspectors - Explain how to initiate and manage the communication process inside and to the outside of the study site team - Acknowledge the need for quality assurance, quality control, and adequate staff competence documentation - Explain the training requirements and documentation in a clinical study - Understand the roles and responsibilities of Scientific Advisory Boards, Data and Safety Monitoring Boards, Data Review committees, etc. | 4 |
| Financial and contractual study management | - Investigator contract - Calculation of investigative site budget - Patient compensation and travel expenses - Invoicing | - Explain the key elements of an investigator contract and negotiation options - Understand the need to define the publication rules in investigator contracts for mono- and multi-centre studies - Describe the elements, calculation process and payment condition options for a site budget - Explain how to calculate and handle subject compensation and travel expenses - Acknowledge the investigator’s role in the site’s invoicing process | 2 |
