# Supplementary material for: A European approach to clinical investigator training
Source: Front Pharmacol. 2013 Sep 9;4:112. doi: 10.3389/fphar.2013.00112 (PMC3766792; doi:10.3389/fphar.2013.00112)
Supplement: Supplementary file 3 [file DataSheet3.DOC]

**Table 3 Clinical Investigator Training – Level 3**

Contents and learning outcomes of Level 1 (Table 1) and Level 2 (Table 2) +

| **Topic** | **Contents** | **Learning outcomes** | **Duration**  **(hrs)** |
| --- | --- | --- | --- |
| Medicines discovery, develop-ment, optimisa-tion and observation | - Medicines discovery - Pre-clinical development - Exploratory and confirmatory development - Marketing authorisation for medicinal products in the EU - Pharmacovigilance in medicines development - Off-label use of medicines - Treatment optimisation - New indications and patient populations - New galenic formulations - Health economics - Pharmaco-epidemiology | - List current challenges and opportunities for medicines development - Outline the discovery process - Understand the importance and principles of toxicology studies - Describe concept and elements of exploratory and confirmatory medicines development - Explain the principle regulatory options for marketing authorisation in the EU - Describe the principles of pharmacovigilance in medicines development - Understand when and why to develop a risk management plan - Explain the problems of off-label use - Define the principles of treatment optimisation studies - Describe the regulatory process and types of studies required to achieve marketing authorisation for a new indication or patient population - Explain the need and general content of a Paediatric Investigation Plan (PIP) - Understand the key technical and regulatory requirements for the development of a new galenic formulation of chemical and biological products - Outline the types of studies required to answer health economic questions - Explain the types of studies performed in pharmaco-epidemiology and their regulatory environment | 3 |
| Sponsor responsibilities | - Overall study responsibility - Scientific and ethical responsibilities - Regulatory responsibilities - Organisational responsibilities - Quality responsibilities - IMP responsibilities - Contractual responsibilities - Insurance responsibilities - Financial responsibilities - Obligations to the public | - Recognise that the sponsor is ultimately responsible for all aspects of the study and for ensuring compliance with the protocol and all applicable regulations - Explain the options and challenges of co-sponsoring in multi-national trials - Explain the sponsor’s responsibility for selection of scientifically and ethically relevant study objectives and suitable study design to answer the scientific question - Define the approvals/licences/authorizations required in the EU and the investigator’s country when conducting interventional and non-interventional studies - Define the criteria that must be met for a trial to be described as non-interventional - Identify the time points when communication with regulatory authorities and ethics committees is required - Explain the process of CTA approval and substantial amendments - List the content of a CTA submission dossier in the investigator’s country - Explain the process and requirements for achieving a favourable ethics committee opinion in the investigator’s country for the study and substantial amendments - Describe the sponsor’s safety reporting obligations - Describe the sponsor’s reporting obligations to competent authorities and ethics committees during and at the end of the study - Explain the sponsor’s obligations for site selection including its capacity and qualification - Recognize the sponsor’s obligation to ensure patient and data protection, unbiased data evaluation, final reporting and publication - Describe the principles of quality management and its applicability to an investigative site - List the types of contracts a sponsor needs to have in place before starting the study - Acknowledge the sponsor’s obligation to have suitable product liability and patient liability insurance in place - Recognize the sponsor’s obligation to have sufficient financial means to perform the study | 4 |
| Human pharma-cology | - Non-clinical requirements for First-in-Human studies - Calculation of the first dose in man - Principles of First-in-human studies - Mechanisms of **A**bsorption, **D**istribution, **M**etabolism and **E**limination - Bioanalytics - Pharmacokinetics and pharmacodynamics - Bioavailability and bioequivalence - Interaction studies - Genetic testing | - Understand the specificity and risk factors of a First-in-human study - Understand the main pre-clinical requirements for studies in humans as described in the ICH-M3 guideline - Define the basic concepts for calculation of the first human doses (NOAEL, MABEL…) - Describe choice of subjects, possible dose escalation schemes, stopping rules, set-up and and precautions to be applied in human pharmacology studies - Understand the conditions for reliable sample collection, work-up, storage, shipment, analysis, and reporting of biological data - Understand the main pharmacokinetic parameters - Explain the principles of bioavailability and bioequivalence - Describe concept and need for interaction studies - Understand the concepts of genetic characterisation of study participants | 1 |
| Concepts for Phase II and III studies | - Research questions - Primary and secondary objectives - Primary and secondary parameters - Design of the study - Treatment duration - Assessments and procedures - Statistical concepts - Study population | - Describe the process required to define a suitable research question including the concept of equipoise - Acknowledge the relevance and suitability of drug-related guidelines - Identify appropriate primary and secondary objectives and parameters to answer the research questions - Explain various study designs and their strength and weaknesses in terms of internal and external validity for the different phases in medicines development - Define the criteria for deciding on the treatment duration in a study - Acknowledge the conflict between statistically required sample size, study conduct practicalities and available budget - Recognize the need for limiting the number of visits and assessments / procedures per subject to enable efficient and timely study performance - Recognize main types of analysis (continuous outcomes, binary outcomes, survival analysis, multivariate analyses) and which applies to a given outcome - Understand the concept and implications of interim analyses - Explain the concept and practical challenges of adaptive designs - Describe the principle and benefits of stratification - Define the different types of analysis populations and the need for a blind data review process - Understand the need for clearly defined inclusion criteria and the detrimental effect of too many and too narrow exclusion criteria | 3 |
| Outcomes and comparator in a clinical study | - Types of outcomes - Outcomes evaluation - Tools for reduction of variability - Types of comparison - Placebo - Active comparator - Blinding options | - Define “outcome study” and “surrogate endpoint” - Recognize the need to identify outcomes that are relevant to patients - Understand the importance and complexity of collection of quality of life data and patient reported outcomes - Define biomarker and the criteria for using a biomarker as surrogate endpoint - Acknowledge the need for extensive training and supervision in outcomes with potentially large inter-reader variability - Describe the advantages and disadvantages of centralised collection and reading of data (e.g., central ECG or image reading, central lab, central event review, DSMB, etc.) - Define the concept of “comparison” in interventional and non-interventional studies - Identify situations where placebo is scientifically required and ethically acceptable - List criteria for a comparator to be acceptable - Describe the options, logistic complexities and regulatory requirements for blinding study medication | 1 |
| Writing a protocol | - Structure of the protocol - Elements of the protocol - Development and review process - Protocol authorisation process - Protocol modification | - Acknowledge the need for a protocol for all types of studies in humans - Describe the structure of the protocol including the relevance of a summary, the background description, and the instructions for study physicians - List the key contributors and stakeholders when developing and reviewing a protocol - Describe tools and procedures to increase the quality of a protocol including patients’ role in this process - Describe the protocol authorisation process and list the required signatures on a protocol - Provide examples of changes to a study that would require a substantial or non-substantial amendment - Describe the format and development process for a substantial amendment | 2 |
| Budget of a clinical study | - Budget elements - Cost calculation process - Funding options - Budget supervision process - Changes to the budget - Final study costs | - Define the complete list of budget elements in a clinical study - Describe the process of cost calculation in a clinical study - Differentiate the routine care costs and the study costs - Understand the financial rules in public hospitals - Describe funding options for sponsor-investigators and related application processes - Explain the options and conditions in public-private partnerships - Describe the principles and tools of budget supervision including the concept of “cost versus budget comparison” - Explain conditions and processes involved in calculating and approving budget changes - Understand the process of final cost   consolidation and reporting | 2 |
| Clinical study manage-ment | - Feasibility - Selection of participating countries and sites - Clinical study team - Assignment of responsibilities - Project management - Study initiation - Monitoring - Reporting - Risk management | - Explain the process for obtaining reliable feasibility information - Describe the criteria and process for selection of participating countries and sites - Explain the management structure in a clinical study - Acknowledge the need for project management resources, skills and tools as well as the benefit of preparing a project management plan that includes a detailed risk analysis - Acknowledge the benefits of preparing manuals in multi-centre studies for crucial processes like monitoring manual, IMP manual, CRF manual, safety data manual, etc. - Explain how to estimate the required human resources to perform all study activities - Describe the concept of outsourcing and tools to identify the suitability of service providers - Explain the process of clearly assigning roles and responsibilities within the project - Explain the process and tools for recruitment projection - List the requirements for study initiation - Explain how the level of monitoring is defined in a risk-based approach - Describe process, advantages and disadvantages of central monitoring - Describe how to calculate the resources required for monitoring - Define the format for monitoring reporting, the process for the monitoring reports’ review and for initiation of corrective actions - Explain the process for reporting on study progress - Describe the options for early risk identification and mitigation - Describe measures to rescue a study that is delayed and/or over budget | 3 |
| Study medication | - Definitions - GMP conformity of study medication - IB - SPC | - Explain the criteria for defining study medication, baseline treatment, rescue medication, concomitant medication, etc. - Explain the types of study medication the sponsor is obliged to provide for free - Understand the requirements for GMP-conform manufacturing, labelling and packaging of study medication - List the situations when a sponsor needs to work with GMP-certified pharmacies at the investigators’ sites - Describe the content of an Investigator Brochure for non-authorised medicines - Explain the content of an SPC - Recognize the sponsor’s responsibility for drug reconciliation | 1 |
| Pharmaco-vigilance in a clinical study | - SUSAR - DSUR - Early study termination - External safety supervision | - Explain the assessment process from SAE to SUSAR - Describe the expedited SUSAR reporting process and timelines in national and multi-national studies - Define the process and timelines for preparation of Development Safety Update Reports (DSURs) - Explain how to calculate the resources for the pharmacovigilance process Identify the reasons why a trial can be stopped (efficacy, safety concern, futility) - Explain conditions requiring external safety supervision in a clinical study | 1 |
| Data manage-ment and Statistics | - CRF preparation - Data management process - Statistical Analysis Plan - Statistical evaluation | - Define the documents that will be used to create the structure of the CRF - Explain the criteria for selecting a paper or electronic CRF approach and the respective implications for data base programming, study management and data cleaning process - Describe the data management process from CRF preparation, to validation, completion, and cleaning until data base lock - List the procedures that can be used to ensure that high quality data are reported in the CRF - List the identifiers that must be on every page of the CRF - Explain how to calculate the resources required for the data management process in a study - Describe the content of a Statistical Analysis Plan and the process of its generation - Define process and timelines for preparation of the statistical evaluation - Recognize the importance of a clearly defined database lock - Understand the difference between per-protocol analysis and post-hoc analyses - Explain the statistician’s role in preparation of the Clinical study report | 1 |
| Documen-tation, reporting and archiving | - Trial Master File - Investigator Site Files - Archiving - Clinical study report - Result communication | - Explain the need and content of a Trial Master File (TMF) - Acknowledge the archiving responsibilities of a sponsor - Describe archiving tools and processes - Identify the needs and requirements for clinical study reports - Describe the content and structure of a clinical study report - Understand the process of critical review of the report - Acknowledge the importance of results communication to participants | 1 |
| Quality manage-ment | - Auditing - SOPs - Training - Qualification documentation | - Explain how to define the extent of auditing - Describe the course of a typical audit and its potential outcomes - Prioritise the need for SOPs in different clinical study aspects - Acknowledge the sponsor’s responsibility for training of all resources - Define the required extent, process and documentation of training for the different stakeholders in a clinical study - Explain the need for proper documentation of qualification within a clinical study by CVs related to defined job descriptions | 1 |
